# Supplementary figures and images for: C-terminal tagging, transmembrane domain hydrophobicity, and an ER retention motif influence the secretory trafficking of the inner nuclear membrane protein emerin
Source: eLife. 2025 Aug 28;14:RP105937. doi: 10.7554/eLife.105937 (PMC12393879; doi:10.7554/eLife.105937)

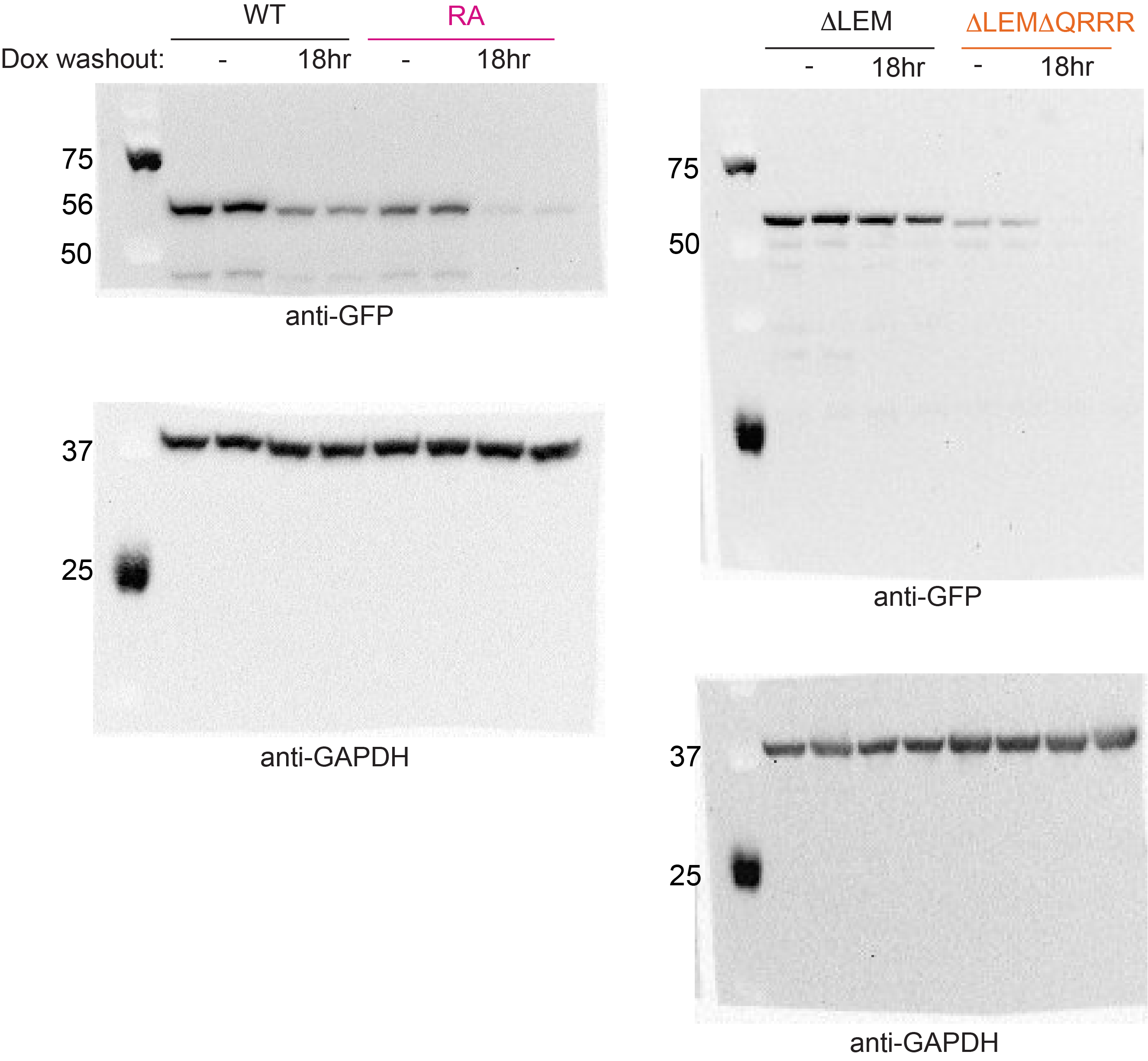

Supplement: Figure 2—source data 2. [file elife-105937-fig2-data2.zip › Figure 2-source data 2/Figure 2 source data annotated.png]

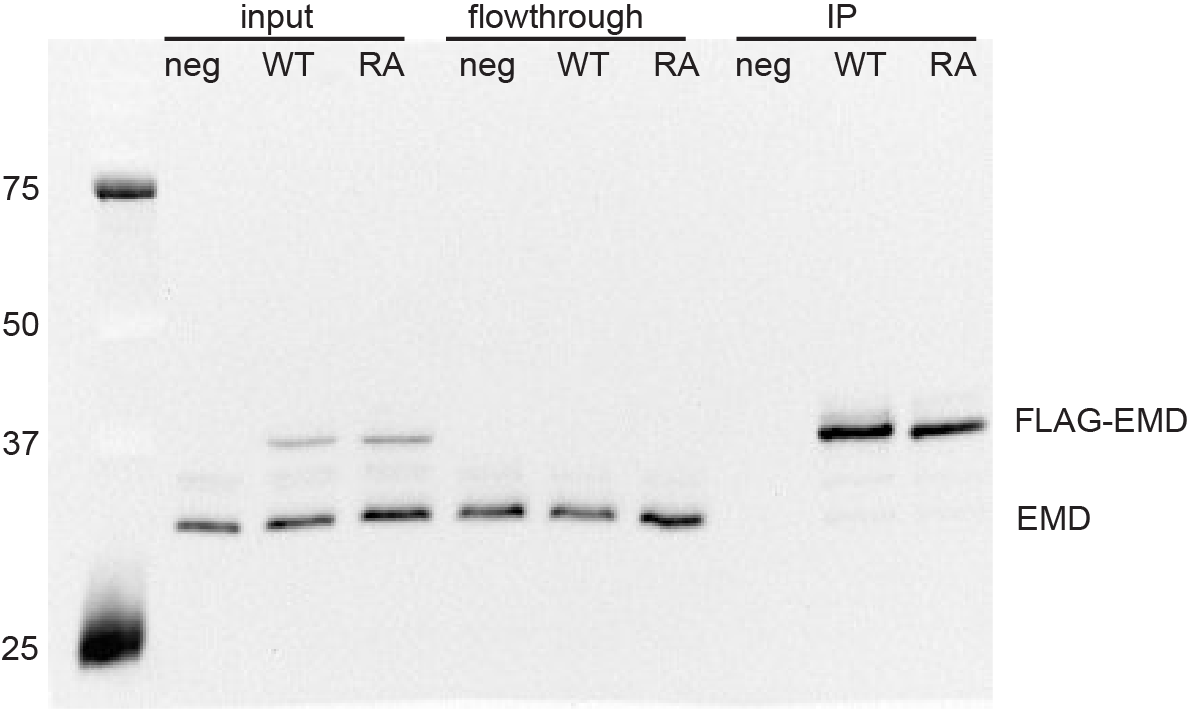

Supplement: Figure 3—figure supplement 1—source data 2. [file elife-105937-fig3-figsupp1-data2.zip › Figure 3-figure supplement 1- source data 2/Figure 3 supplement 1 source data annotated.png]

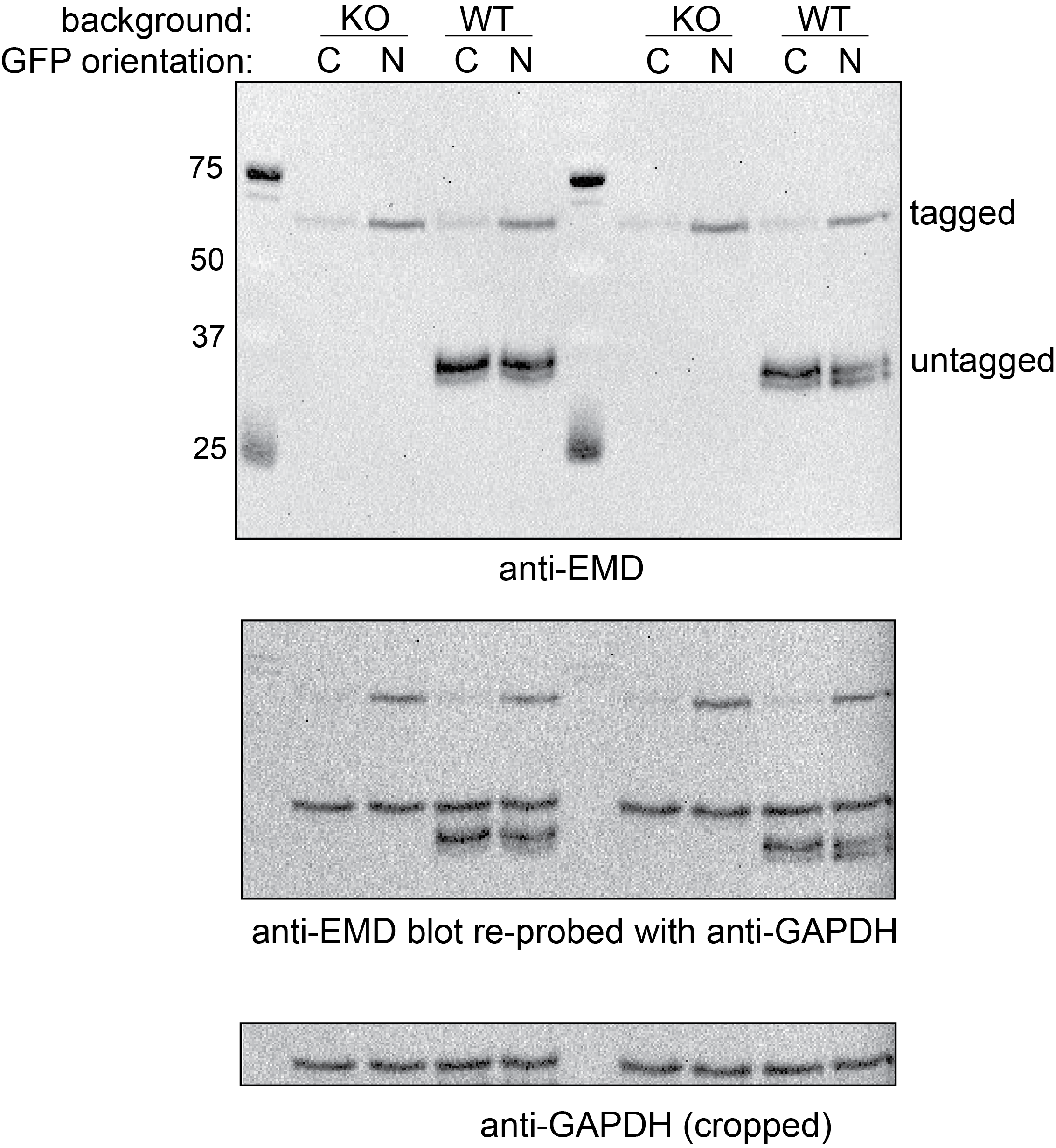

Supplement: Figure 4—source data 2. [file elife-105937-fig4-data2.zip › Figure 4-source data 2/Figure 4 source data 2 annotated.png]
